# A Coproduction Community Based Approach to Reducing Smoking Prevalence in a Local Community Setting

**DOI:** 10.1155/2016/5386534

**Published:** 2016-06-30

**Authors:** G. J. McGeechan, D. Woodall, L. Anderson, L. Wilson, G. O'Neill, D. Newbury-Birch

**Affiliations:** ^1^Institute of Health and Social Care, Teesside University, Constantine Building, Borough Road, Middlesbrough TS1 3BA, UK; ^2^Children and Adult Services, Public Health, Durham County Council, Durham DH1 5UJ, UK

## Abstract

Research highlights that asset-based community development where local residents become equal partners in service development may help promote health and well-being. This paper outlines baseline results of a coproduction evaluation of an asset-based approach to improving health and well-being within a small community through promoting tobacco control. Local residents were recruited and trained as community researchers to deliver a smoking prevalence survey within their local community and became local health champions, promoting health and well-being. The results of the survey will be used to inform health promotion activities within the community. The local smoking prevalence was higher than the regional and national averages. Half of the households surveyed had at least one smoker, and 63.1% of children lived in a smoking household. Nonsmokers reported higher well-being than smokers; however, the differences were not significant. Whilst the community has a high smoking prevalence, more than half of the smokers surveyed would consider quitting. Providing smoking cessation advice in GP surgeries may help reduce smoking prevalence in this community. Work in the area could be done to reduce children's exposure to smoking in the home.

## 1. Introduction

Public health initiatives tend to be complex and context specific and it is essential that they are evaluated to prove effectiveness. However, most evidences informing public health policy tend to be in the form of tightly controlled, intervention trials conducted by universities which raises questions around the transferability of research to “real world” practice [1]. Whilst many see researchers from academia and public health practitioners as coming from two different worlds, the boundaries between them are often smaller than many believe [2]. A coproduction approach to health initiatives involving researchers and public health practitioners working together could lead to evidence which is more translational into real world practice [3–5]. In addition to researchers and practitioners working together to evaluate services, coproduction can benefit from engaging communities in research and evaluation as this allows service users to become equal partners in service provision and allows practitioners to become facilitators of a service that concentrates on the skills and abilities of the local community [6, 7].

The traditional method of delivering services to improve health is based on meeting needs or delivering treatment. Experts are parachuted into communities that are defined by their perceived deficiencies, referred to as “areas of multiple deprivation” or “areas of high crime,” to offer treatment to individuals characterised as “smokers,” “alcoholics,” or “drug addicts.” Dropping in services to sort out community problems takes away control from the communities themselves and makes them passive recipients of services [8]. However, no matter how deprived a community is perceived to be, every community has assets, which are the collective resources individuals and communities have at their disposal which can protect against negative health outcomes; these assets can be financial, physical, environmental, and even the people within the community itself [9]. By working with communities, it is possible to develop services which utilise all of these assets, are meaningful to local people who will access them, and help to protect against adverse circumstances, thereby promoting health and well-being [8, 10].

There are many different names for coproduction research such as knowledge translation, participatory action research, and collaborative research, which can vary greatly in terms of methods depending on the area of interest. However, most tend to adhere to similar principles with the exchange, synthesis, and dissemination of knowledge between researchers or policy makers and end users seen as key [3]. The Canadian Institutes of Health Research adheres to the knowledge to action framework, which was developed by Straus and colleagues [11] which can be utilised at all levels of translational research from the local level to the global level. Within this model is a clearly defined process for knowledge creation which may be useful for asset-based health research consisting of three key phases;* knowledge inquiry; synthesis of knowledge, and creation of knowledge tools*. Knowledge inquiry includes the completion of primary research, whilst synthesis of knowledge includes bringing together research findings relevant to the topic. The final stage involves further synthesis of the best quality knowledge into decision making tools, or policy and practice guidelines, or delivery of services [11].

This paper outlines some baseline results of an evaluation of a coproduction, asset-based approach to improving health and well-being within a small community in the North East of England. Public Health England (PHE) local health profiles highlights that this area suffers from high levels of income deprivation, high levels of unemployment, and poorer health outcomes such as higher incidences of lung cancer and chronic obtrusive pulmonary disease when compared to the rest of England [12]. The relationship between social deprivation and health outcomes is complicated and is affected by a number of factors, such as low educational attainment, being unemployed or working in a routine/manual occupation, low income, and ethnic background [13].

Evidence has shown that these factors may contribute to unhealthy behaviours such as the use of illicit drugs [14], alcohol consumption [15], and smoking cigarettes [16]. In the United Kingdom (UK), the number of smokers has been steadily declining since the 1970s; however, around 18.5% of the adults in the UK smoke cigarettes, [17]. Evidence suggests that socioeconomic factors may be associated with smoking behaviour with men and women from routine or manual backgrounds being three times more likely to smoke than those from managerial or professional backgrounds [17, 18].

Smoking and exposure to second-hand smoke can have serious implications for health with the link between smoking and lung cancer long accepted [19]; smoking can also increase the risk of coronary heart disease [20] and exacerbate the symptoms of asthma [21]. Evidence suggests that exposure to second-hand smoke is more dangerous for children, who are more susceptible to the pollutants associated with second-hand smoke. As such, they are at an increased risk of lower respiratory infections, meningococcal diseases, and exacerbated symptoms of asthma [22]. Early exposure to second-hand smoke also increases the likelihood that children will take up smoking later in life [23]. The UK government has recently introduced smoke-free legislation making it illegal to smoke in a car when a child is present to try and reduce the risk [24]. However, whether this will have any impact on children's exposure to second-hand smoke in the home remains to be seen.

This service to be evaluated was led by a consortium of health service providers who were commissioned to identify and promote the assets within a community in order to develop services which fit local need. Whilst this programme of work involves many different elements, this paper focuses on the knowledge inquiry phase which involved the development of a community led survey which aimed to ascertain smoking prevalence within the community, as well as examining attitudes towards smoking cessation, smoking in public places, and smoking in the presence of children.

## 2. Methods

### 2.1. Phase One: Recruitment of Community Volunteers

In order to develop a coproduction approach to health improvement, it is important to engage with members of the community [7]. The first step in this process involved the service providers engaging with members of the community who were interested in volunteering as community researchers. An independent researcher undertook an asset mapping exercise where they spent time in the community getting to know who held prominent positions within the community and were likely to be held in high regard by other residents [25]. As the asset mapping exercise was independent from the evaluation, the results are not reported within this paper. After the completion of the asset mapping, those individuals who held a key role within the community were approached by the service providers and asked if they would be interested in becoming community researchers. This coproduction approach to service delivery provided the volunteers with an opportunity to develop useful skills and provided the evaluation team with access to local residents who could complete the survey.

Once individuals had been identified, a number of training sessions were carried out to enable them to become community volunteer researchers who would conduct the baseline survey. Two members of staff from the service provider also attended training to provide support in the case that community researchers had difficulty in recruiting participants. The training comprised a 2-hour session conducted by one representative from the university, and one representative from the local authority and consisted of training around obtaining informed consent, the need for confidentiality, and how to conduct the survey without leading participants towards any particular answer. As part of the training sessions, volunteers had the opportunity to practice the survey to get used to how it is filled out and to go over any questions that they or participants may have. A “how not to conduct a survey” session was also run to give the volunteers an idea of bad practice and things they should try to avoid. Volunteers received no monetary compensation for delivering the survey but were presented with a certificate from the university in recognition of completing their training and were made aware of other opportunities to participate with the service within their local community.

### 2.2. Phase Two: Smoking Survey

In order to assess smoking prevalence in the local area and understand people's attitudes towards smoking and their knowledge of local stop smoking services, a survey was distributed to a random sample of the local population between December 2014 and February 2015. A follow-up survey will be administered in May 2016 to assess the ongoing impact of this programme of work on smoking prevalence and attitudes towards smoking cessation. The survey used in this evaluation was adapted from a similar study which was carried out in a deprived area of Northern England; this particular area had a smoking prevalence rate of 45.0%, and we would expect there to be a similar prevalence within our community [26].

### 2.3. Recruitment

A sample size calculation was carried out which identified that in order to detect a drop in smoking prevalence of 10.0% between the two time points a sample of 376 participants was required at each time point. A convenience sampling technique was used to recruit participants into this study. Volunteers were asked only to recruit participants into the study whom they naturally came into contact with in their various roles within the community. Volunteers were discouraged from knocking on people's doors or from entering business premises to recruit individuals. However, the two members of staff from the service providers had more flexibility to recruit participants as they were covered by their organisations' lone worker policy and could therefore recruit from other areas of the community.

### 2.4. Data Collection and Analysis

Data was collected via a smoking survey which was delivered in paper format in the local community by the volunteer community researchers and service providers. The survey was adapted, with permission from a similar survey which was delivered in another area of the North East of England. Questions in this survey were drawn from a variety of sources, such as the Wreckenton Household Survey [26], the annual Office for National Statistics (ONS) Smoking Attitudes and Behaviour Survey [27], and the ONS Personal Well-Being questions [28]. Additional questions were added to the survey and were agreed upon by the project steering group. The survey measured the following variables.

#### 2.4.1. Smoking Prevalence

Participants were asked a number of questions relating to whether they were a current smoker, how much they smoked, where they buy their cigarettes from, and whether they have used e-cigarettes. Descriptive statistics were used to highlight the smoking prevalence of the community and a series of Wilcoxon Signed Ranks Tests were used to highlight differences in the frequency distribution of responses.

#### 2.4.2. E-Cigarettes

Two questions were used to assess the prevalence of smokers who have tried an electronic cigarette. Participants were asked if they have ever used an electronic cigarette and for what reason they had tried an electronic cigarette. Descriptive statistics were used to highlight the proportion of smokers who had and had not tried an e-cigarette and to illustrate for what reasons smokers were using e-cigarettes. A Wilcoxon Signed Ranks test was used to illustrate the frequency distribution of smokers who had used an e-cigarette, grouped by their intention to quit.

#### 2.4.3. Quitting Intentions

Three questions were used to assess attitudes towards smoking cessation. Participants were asked whether they were considering quitting smoking, how much they would like to quit smoking, and whether or not they had attempted to quit smoking in the previous 12 months. A number of Wilcoxon Signed Ranks Tests were conducted to look for differences in frequency distribution of answers to the first two questions, a crosstabs contingency table was used to illustrate the frequency distribution of responses to the question around quitting attempts, and a Pearson's chi-squared analysis was conducted to see if there were any significant differences in the frequency distribution of responses.

#### 2.4.4. Children's Exposure to Second-Hand Smoke

Four questions were used to determine children's exposure to second-hand smoke. Participant's smoking status and whether or not anyone else in their house was a smoker were used to identify smoking households. Participants were then asked how many children under the age of 18 lived in their house. Finally, participants were asked what the rules were for smoking in their home when a child was present. Descriptive statistics were used to illustrate the proportion of children living within a smoking household and those directly exposed to second-hand smoke.

#### 2.4.5. Well-Being

Four questions were asked to measure the personal well-being of local residents; these questions were designed by the ONS to measure people's thoughts and feelings about their own quality of life [28]. All questions were measured using a 10-point Likert scale where 1 indicated not at all and 10 indicated completely. Participants were asked how satisfied they are with life nowadays; to what extent they you feel that things in their life are worthwhile; how happy they felt yesterday; and how anxious they felt yesterday. A series of *t*-tests were used to look for differences in the mean scores for each of the four questions split by their smoking status.

## 3. Results

### 3.1. Phase One: Community Volunteers

Three training sessions were conducted with members of the community who had been identified via the asset mapping exercise. The training session lasted for 2 hours and consisted of training around how to deliver the survey, the importance of confidentiality, and how to gain verbal consent from participants. Over the three sessions, ten community volunteers were trained to deliver the survey, and two members of staff from the service providers were also trained. A summary of researcher characteristics can be seen in Table 1.

### 3.2. Phase Two: Smoking Survey

#### 3.2.1. Participants

A sample size calculation was conducted which identified that 376 participants needed to be recruited in order to detect a 10% drop in smoking prevalence over time. A total of 228 surveys were completed by local residents (60.1%). Of those who completed the survey, 94 (41.2%) were male and 127 (56.6%) were female; participants were predominately White British (96.5%). When looking at employment status, more participants were employed (34.2%) than unemployed (25.4%) or retired (33.3%); however, these differences were not significant. A summary of participant characteristics is presented in Table 2.

#### 3.2.2. Smoking Prevalence

**Figure 1:**
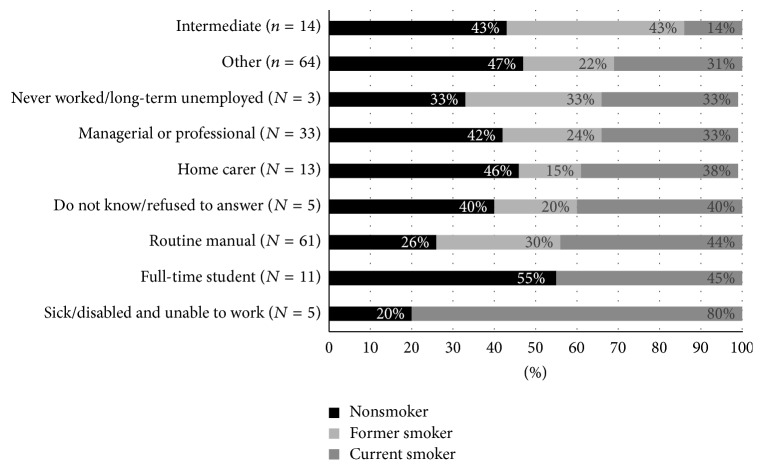
Breakdown of smoking status by occupation (*N* = 209).

Of the 228 respondents, 82 (36.0%) were identified as current smokers, 54 (23.7%) were identified as former smokers, 89 (39.0%) stated that they had never smoked, whilst three participants (1.3%) did not answer this question.  Table 3 and Figure 1 illustrate the smoking status of participants broken down by age, gender, and occupation. When looking at smoking status by the age of respondent, a significant association was observed using a Fisher exact probability test, (*p* < 0.05) with a higher proportion of former smokers being over the age of 45. No other significant differences were found. In addition to this, respondents were asked to indicate how many people in their household smoked. Of the 143 participants who either had never smoked or were former smokers, 31 indicated that they lived in a house with at least one smoker. This suggests that around 48.7% of all respondents within the community live within a smoking household.

#### 3.2.3. E-Cigarette Use

Of those who identified themselves as smokers, 51.9% indicated that they had tried an e-cigarette. Participants were then asked to indicate the reasons why they had used an e-cigarette, of the 41 participants who have used an e-cigarette, 32 (78.0%) answered this question. The most common reasons reported by participants as to why they had tried an e-cigarette was to help them stop smoking entirely (37.5%), to reduce their smoking (34.4%), as a stop smoking aid (18.8%), and as a smoking substitute (9.4%).  Table 4 shows the quitting intentions of smokers split by e-cigarette use; this illustrates that people who were not considering quitting smoking were less likely to have used an e-cigarette than those who were trying to quit smoking. However, this difference was not statistically significant.

#### 3.2.4. Quitting Intentions

Participants' quitting intentions were measured using three items on the survey. No differences were observed between quitting intentions and the age and gender of participants.

Firstly, participants were asked to indicate whether they were currently considering quitting smoking; a total of 21.9% of smokers indicated that they were currently trying to quit smoking, 12.2% of smokers indicated that they were considering quitting smoking, 30.5% of smokers are not ready to quit yet, whilst 31.7% of smokers indicated that they had no intention of quitting. Furthermore, 69.5% of smokers indicated that they would like to quit smoking and 45.1% of smokers have made at least one attempt to quit smoking in the 12 months preceding the survey.

Participants indicated that they would consider using a wide range of methods to aid them in smoking cessation, with the most common responses being through a consultation with their GP or local chemist. Furthermore, with recent guidance on e-cigarettes being published by Public Health England, it is interesting to note that people who were considering quitting smoking appeared more likely to have used an e-cigarette than those who are not considering quitting, although this difference was not statistically significant.

#### 3.2.5. Children's Exposure to Second Hand Smoke

Participants were asked to indicate how many children under the age of 18 currently lived in their house and how many smokers were currently living in the household. A summary of responses to these questions can be seen in Table 5. The results indicate that 22.7% of respondents who answered these questions lived in a household with at least one child and one adult smoker. However, when we excluded households where no children lived, 49 out of 75 households had at least one smoker and one child living there. This suggests that 63.1% of children in the area reside within a smoking household. However, when participants from the 49 smoking households were asked what the rules were for smoking in the house when a child was present, 10.2% indicated that they would allow smoking at any time, 38.8% sometimes allow smoking, whilst 49.0% would not allow smoking at all.

#### 3.2.6. Well-Being

**Figure 2:**
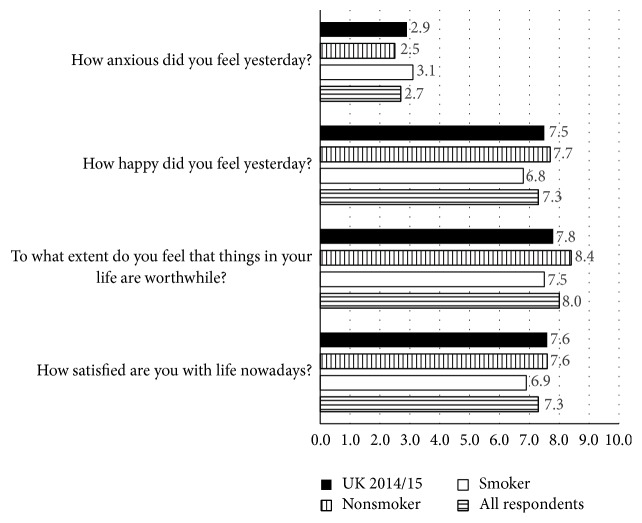
Well-being scores by smoking status.

Participants were asked four questions designed by the ONS to measure personal well-being in the UK.  Figure 2 illustrates that whilst nonsmokers scored higher than smokers on the three well-being questions and lower than smokers on the anxiety question, the differences were marginal and not statistically significant.

## 4. Discussion

Whilst the traditional method of delivering services to improve health is based on meeting needs and delivering treatment, with “experts” parachuted into deprived communities to fix them, research has shown that by working with communities it is possible to develop services which utilise assets and are meaningful to local people [3–7]. As part of a project aimed at using community assets to improve health and well-being, we trained a group of community researchers to deliver a smoking prevalence survey within a small community in the North East of England. The results of this survey will be fed back to the community with the hope that it will help community assets develop meaningful services which could help reduce smoking prevalence.

There certainly appears to be scope to focus efforts in the area on smoking cessation as over 50% of current smokers indicated that they either were trying to quit or would like to in the future. With GP surgeries and pharmacies emerging as the most likely source of smoking cessation support within the community, any efforts to reduce the smoking prevalence in this area should be concentrated there. There has been recent attention on the role of e-cigarettes in promoting smoking cessation [29], with Public Health England recently endorsing their use [30]. Over 50% of smokers in this area had tried an e-cigarette on at least one occasion, with the most common reason for trying one being a stop smoking aid. Whilst there is still some debate about the health implications of e-cigarettes [31], a recent systematic review indicated they may be an effective way of promoting smoking cessation, and with many residents already using them it may be an effective method in this area [29].

Smoking prevalence within this community was 35.9%, higher than both the regional (20.9%) [32] and the national average (18.5%) [17]. Whilst no statistical differences were found, the spread of smokers by age, gender, and occupation was similar to national trends, with smokers most likely to be males, younger adults, and from a routine or manual occupational background [17]. This research highlighted that 48% of adults and 63.1% of children within the community are potentially being exposed to second-hand smoke which can have significant consequences for health [20, 21, 24] especially for children [22]. Whilst only 10.2% of smoking households allow smoking in their house at any time with a child present, research has shown that residual chemicals in furniture also pose a significant risk to health. Therefore, work in the future could focus on highlighting the health consequences of second-hand smoke and the dangers of third-hand smoke, especially to children [33–35].

### 4.1. Limitations

One of the limitations of using convenience sampling is that you may not necessarily get a representative sample [36]. This is compounded when using community volunteers as researchers as they may only recruit participants from within their own social network. However, whilst this may be the case, we feel that those participants who completed the survey represented a broad spectrum of ages, genders, and employment statuses. Whilst all volunteers and 99% of those who completed the survey were identified as White British, this is in line with the population of the local authority area where 95.3% were identified as White British [37].

### 4.2. Next Steps

The results of this survey will be fed back to the local community and to the service providers who will continue to work with assets in the community to develop services which are meaningful to local residents. We will repeat the smoking prevalence survey in 18 months' time to see what impact a community asset-based well-being programme has had on the local smoking prevalence, attitudes towards smoking cessation, children's exposure to second-hand smoke, and uptake of local smoking cessation services.

## Figures and Tables

**Table 1:** Community researcher characteristics.

	*N* = 12	
	Number	Percentage
*Gender*		
Male	5	41.6%
Female	7	58.3%

*Age group*		
18–44	7	58.3%
45+	5	41.6%

*Employment status*		
Employed	8	66.7%
Unemployed	4	33.3%
Retired	0	0.0%
Student	0	0.0%

*Ethnicity*		
White	12	100%
Other	0	0.0%

**Table 2:** Participant characteristics.

	All respondents *N* = 228		Current smokers *N* = 82^*∗*^		Nonsmokers *N* = 143^*∗*^	
	Number	Percentage	Number	Percentage	Number	Percentage
*Gender*						
Male	94	41.2%	39	17.1%	55	24.1%
Female	129	56.6%	42	18.4%	86	37.7%

*Age group*						
18–44	98	43.8%	43	18.9%	54	23.2%
45+	124	54.4%	37	16.3%	87	38.2%

*Employment status*						
Employed	78	34.2%	30	13.2%	48	21.1%
Unemployed	58	25.4%	30	13.2%	28	12.2%
Retired	76	33.3%	18	7.9%	58	25.4%
Student	6	2.6%	2	0.9%	8	3.5%

*Ethnicity*						
White	219	96.5%	79	34.6%	139	60.9%
Other	1	0.44%	1	0.44%	0	0%

^*∗*^3 participants did not indicate their smoking prevalence.

**(a):** 

Smoking status	Aged 18–44	Aged 45+	Total
Current smoker	43 *(44.3%)*	37 *(29.8%)*	80 *(35.1%)*
Former smoker	14 *(14.4%)*	40 *(32.2%)*	54 *(23.6%)*
Never smoked	40 *(41.2%)*	47 *(37.9%)*	87 (38.2%)

**Total**	**97 *(42.5%)***	**124 *(54.4%)***	**221**

**(b):** 

Gender	Male	Female	Total
Current smoker	**39 ** *(41.5%)*	**41 ** *(32.3%)*	**80** *(35.1%)*
Former smoker	**18 ** *(19.1%)*	**36 ** *(28.3%)*	**54** *(23.6%)*
Never smoked	**37 ** *(39.3%)*	**50 ** *(39.4%)*	**87** *(38.2%)*

**Total**	**94 (41.2%)**	**127 (55.7%)**	**221**

**Table 4:** Quitting intentions by use of an e-cigarette (*N* = 77).

Used an e-cigarette?	Quitting intentions			
	Trying to quit	Thinking about quitting	Not ready to quit	Do not want to quit
Yes	13 (72.2%)	2 (20.0%)	17 (68.0%)	8 (33.3%)
No	5 (27.8%)	8 (80.0%)	8 (32.0%)	16 (66.7%)

Total	18 *(100.0*%)	10 *(100.0*%)	25 *(100.0*%)	24 *(100.0*%)

**Table 5:** Children living within a smoking household.

Number of children living in household	Smoking household		Nonsmoking household	
	Number	Percentage	Number	Percentage
*0*	*62*	*28.8*%	*78*	*36.3*%
1	26	12.1%	2	0.9%
2	12	5.6%	17	7.9%
3	4	1.9%	7	3.3%
4	5	2.3%	0	0.0%
5	2	0.9%	0	0.0%

*Total responses*	*111*		*104*	

*Total children*	*92*		*57*	
